# Generalized Unsafety Theory of Stress: Unsafe Environments and Conditions, and the Default Stress Response

**DOI:** 10.3390/ijerph15030464

**Published:** 2018-03-07

**Authors:** Jos F. Brosschot, Bart Verkuil, Julian F. Thayer

**Affiliations:** 1 Institute of Psychology, Unit Health, Medical and Neuropsychology, Leiden University, 2300 RB Leiden, The Netherlands; 2 Institute of Psychology, Unit Clinical Psychology, Leiden University, 2300 RB Leiden, The Netherlands; bverkuil@fsw.leidenuniv.nl; 3 Department of Psychology, The Ohio State University, Columbus, OH 43210, USA; thayer.39@osu.edu

**Keywords:** stress theory, default stress response, chronic stress, generalized unsafety, perceived safety, somatic disease, loneliness, low social status, natural versus urban environment, early life adversity

## Abstract

Prolonged physiological stress responses form an important risk factor for disease. According to neurobiological and evolution-theoretical insights the stress response is a default response that is always “on” but inhibited by the prefrontal cortex when safety is perceived. Based on these insights the Generalized Unsafety Theory of Stress (GUTS) states that prolonged stress responses are due to generalized and largely unconsciously perceived unsafety rather than stressors. This novel perspective necessitates a reconstruction of current stress theory, which we address in this paper. We discuss a variety of very common situations without stressors but with prolonged stress responses, that are not, or not likely to be caused by stressors, including loneliness, low social status, adult life after prenatal or early life adversity, lack of a natural environment, and less fit bodily states such as obesity or fatigue. We argue that in these situations the default stress response may be chronically disinhibited due to unconsciously perceived generalized unsafety. Also, in chronic stress situations such as work stress, the prolonged stress response may be mainly caused by perceived unsafety in stressor-free contexts. Thus, GUTS identifies and explains far more stress-related physiological activity that is responsible for disease and mortality than current stress theories.

## 1. Introduction

Patient: “Doctor, I am scared of the unknown”

Dr. Sigmund: “Such as strange cultures or exotic destinations?”

Patient: “I don’t know, I don’t know anything about it...”

Peter de Wit, strip in De Volkskrant, 14 August 2014

### 1.1. From Stressors to (Un)Safety

Animals including humans are naturally, by *default*, afraid of the unknown [1,2]. As we grow up we learn to recognize the signals of safety, but from the very beginning we fear without a sign of threat—also known as “intolerance for uncertainty” [2,3,4]. Thus, the stress response is always “on”, and it stays on as long as there is no obvious safety. When safety is perceived, the stress response is inhibited. Importantly, to be “on” it does not need a stressor at all: it either remains activated as long as no safety is perceived or is disinhibited when safety disappears. It is a “default” response, meaning that it is a preselected condition to which the system (the organism) falls back when there is no other input i.e., no relevant information, which for the stress response is information regarding safety. The default here is a state of generalized unsafety in which the stress response stays on [1,2]. This may sound strange, and even worse, it seems in stark contrast to current stress theories. Stress theories speak of a stress response as a *response* to a *stressor*, that is, as a direct response to a threat. Yet, this idea of a default stress response determined by safety is firmly based on neurobiological and evolution-theoretical insights that we have explained elsewhere when introducing the novel Generalized Unsafety Theory of Stress (GUTS) [1,2,3]. In the present paper, we will first briefly summarize these insights, and then continue with discussing the implications of GUTS for stress theory. We will specifically focus on its major challenge to account for prolonged physiological stress responses, which is the type of response that is primarily responsible for the disease risk of stress. Essentially, we will argue that by far most of the prolonged responses occur in (chronic) situations *without actual stressors*, and that GUTS can explain this better than conventional stress theories, using the concepts of the default stress response and generalized unsafety.

### 1.2. Neurobiological and Evolution-Theoretical Insights

From the neurobiological perspective, the prefrontal cortex normally suppresses the parts of the brain responsible for the stress response, especially the amygdala [5,6,7,8,9,10]—but only when the brain has perceived safety [4,5,6]. If safety is no longer perceived, the “brake” is immediately lifted, the amygdala resumes its high level of activity and the body is instantly ready to fight or flee away. Heart activity increases and blood pressure goes up among other well-known stress responses. Thus, the stress response is always set to high alertness, but kept inhibited as long as safety can be perceived. Unlike some popular beliefs, neural inhibition costs relatively little energy and is extremely cost-effective, including prefrontal inhibition of the stress response [11]. Removal of prefrontal inhibition (disinhibition) “permits” rather than “causes” the stress response. This neurobiological principle of *letting go* or *release* rather than *turn on* or *push into action* is actually very common and has been known as the *Hughlings Jackson principle* of “hierarchical integration through inhibition” [12] since the 1880s, albeit overlooked by many since then. Importantly this principle implies that the removal of inhibition “permits” rather than “causes” an increase in physiological activity (disinhibition). Releasing phylogenetically old and vital response mechanisms such as the stress response happens much quicker than turning them on. We now know that the prefrontal cortex also suppresses many other predominant, often “impulsive” subcortical responses (see [13]), and many other primary psychobiological functions, such as hunger and immunity, and many behaviors (motor functions), are also regulated in a similar “default-inhibitory” fashion [14,15,16]. Thus, starting immediately after birth, the young animal or human gradually learns the signals of safety, a process that is slowed down but never stops during adulthood.

The default stress response that can be quickly unleashed also corresponds to modern evolution-theoretical insights about stress and anxiety. In the wild, organisms have survived not by waiting for more evidence of threat but instead by erring on the side of caution (e.g., [17,18]), or popularly formulated, to be “better safe than sorry”. It is better to play it safe and to flee 10 times too often than once too few. Those who fled at the first sign of unsafety continued to live and pass their genes [17,18]—especially those spending minimum energy on this default-inhibitory survival mechanism. The default stress response is thus phylogenetically very old and similar across many species, and there is no reason to ignore its presence in humans.

### 1.3. The Consequences of GUTS for Stress Theory: An Overview

The viewpoint that safety perception (or the lack thereof) is the primary determinant unleashing default stress response has several important consequences for the role of the environment in the link between stress and health, as we will discuss in more detail below. Briefly, the first consequence concerns the explanation of the occurrence of the type of stress responses that can cause disease, namely prolonged or chronic stress-related physiological activity. This is—in our opinion—the basic problem of the science of stress and disease. Conventional stress theory has been unable to fully explain this critical mediator of the stress-disease relationship, largely because these theories have remained focused on stressors, that is, actual threats in the environment. However, stressors are not necessary for the stress response to be released: a lack of information about safety is sufficient. With no such information, generalized unsafety is assumed by the brain, and the default stress response is disinhibited. The question should not be “what causes prolonged activity?” but “what stops it”. Accounting for the role of safety and the default quality of the stress response requires a considerable modification and reformulation of the conventional stress theory. The newly proposed theory, GUTS, can explain the presence of prolonged stress-related physiological activity in a range of situations without stressors.

A second important consequence of the notion of the stress response being a default response is that it is largely unconscious, because of its phylogenetic age, its pervasiveness across the animal kingdom, its often continuous nature, and the fact that ontogenetically it is largely determined during prenatal and early life stages to which we have no conscious access.

Finally, a third consequence consists of the astonishing array of situations or conditions (called domains in GUTS terminology) in which prolonged stress response occur *without any stressors*, assumedly because these domains are *compromised* in terms of unsafety. By focusing on stressors stress science seems to have missed a lot. For example, loneliness, prenatal maternal stress, post-natal adversity, and chronic anxiety (including anxiety disorders) are associated with prolonged physiological activity and are now being recognized as belonging to the strongest psychological predictors of organic disease [19,20,21,22,23,24,25,26,27,28,29,30,31,32,33,34,35,36,37]. Purportedly, the reason that these domains were largely missed by stress science is that they cannot easily be understood in terms of stressors or at least no concurrent stressors. Interestingly, some theorists even oppose including anxiety as a stress phenomenon, as being too “global” [38], while in neuroscience, stress and anxiety are commonly treated as the same subject, because of their shared neurobiology (e.g., [5,6,7,8,9,14,39]).

Importantly, many of the domains that we will discuss that are compromised in terms of perceived safety are in fact potential sources of safety, such as a strong social network for humans and other social animals, but also potentially safety promoting environmental factors such as natural surroundings, shelter, and perceptual overview, that are likely to be not only important for animals but for humans too. We will also explore how the “internal environment”, that is, the state of the body, might be crucial in providing signals of safety. Finally, we will argue how stressor-focused science has also missed the very aspects that cause prolonged stress responses in many chronic stressors that it has been studying for a long time.

The next chapter will start with the basic problem of stress and health science that the conventional theories appear not to be able to address well: prolonged stress-related physiological activity.

## 2. The Basic Challenge of Stress Science: Explaining Prolonged Stress-Related Physiological Activity

That basic problem for stress and health science is to explain prolonged physiological stress-related activity, or briefly prolonged activity. Although stress science has historically mainly focused on stress responses *during* stressors, most stress scientists will agree either explicitly or implicitly that for a stressor to lead to disease the stress response to it should be prolonged or sustained [40,41,42,43,44,45,46,47,48,49,50]. A prolonged physiological response will finally lead to a pathogenic state of bodily “wear and tear”, often discusses as allostatic load [45] that will lead to disease. However, as we have argued several times [1,2,48,49,50] stressors are mostly short-lived and typically account for only a tiny fraction of daily stress responses. Generally during their occurrence stressors yield stress responses that are too brief to be threatening to bodily health. Even the events that comprise chronic stressful conditions such as work stress, financial problems, marital discord, and long-term care giving for a sick family member, are on themselves too brief: for example, stressful encounters with a superior, or anger-provoking dealings with one’s marital partner, or unpredictable moments of distress with an Alzheimer patient at home. Still, all these chronic conditions are known to be associated with prolonged activity, often stretching out into leisure time (in case of work stress) and even during sleep ([47,51,52,53,54,55,56]; see for some inconsistencies for some types of work stress [57,58]), and they all carry greatly enhanced risks for somatic disease. For example high work stress quadruples one’s chances of developing cardiovascular disease, the risk for heart problems is threefold in those with problematic marriages and caring for a partner with Alzheimer doubles cardiac risk (see e.g., [59,60,61,62,63,64,65]). These risks are comparable with or even higher than those of smoking and obesity and other classical cardiovascular risk factors. Most importantly, the risks of these chronic stressful conditions are believed to be caused by prolonged physiological activity. However, how to explain this prolonged activity? This is still a major challenge for traditional stress science.

### 2.1. Current Explanations of Prolonged Activity

First, prolonged stress-related physiological activity—briefly called prolonged activity—is not only manifested in slow *recovery* after stressors. Slow recovery is only one aspect. At least as important is *anticipatory* activity to stressors, often far in advance of them. Previously, we hypothesized that *thinking* about stressors, or more formally “cognitively representing stressors”, is responsible for prolonged activity. We called this *perseverative cognition*, with as its primary manifestations worry and rumination. Perseverative cognition was held responsible not only for the anticipatory effects and slow recovery, but also for *recurrent* activity—in between stressors—when we worry or ruminate about past or future stressors [48,49,50]. There is ample evidence now that perseverative cognition affects cardiovascular, autonomic, and endocrine nervous system activity, which together supports the notion that this is a crucial pathogenic pathway from chronic stress to long-term disease outcomes [66]. Later we extended the perseverative cognition hypothesis by proposing that we are *not aware* of a large part of these cognitive representations, and we have called thus “unconscious stress” [67,68]. For this extended theory there is also supportive evidence. Indirect evidence comes from studies that have shown that cardiac activity due to stressors and worry during the day was still enhanced during nocturnal sleep [51,69]—during which conscious worry is not possible. Similarly, autonomic effects during sleep have been observed in subjects who anticipated giving a public speech in the early morning [70]. These effects were not due to dreaming, REM sleep or micro-awakenings and even not to subjective sleep quality. We also found that in daily life, worry itself has prolonged cardiac effects up to two hours after worry episodes [71], independent of mood and life style factors. Thus, during sleep—when one cannot consciously worry—as well as during daily life worrying seems to continue in an unconscious fashion, along with its physiological effects. As yet, there is also growing *direct* evidence that subliminal (i.e., under the awareness threshold)stressful stimuli increase activity in several physiological parameters (see reviews [67,68,72] and recently [73,74], but see [75]), but attempts to measure unconscious stress and show associations with physiological response in the lab and in daily life have yielded varied results, depending on the type of measurement (see [67,68] and recently [73,76,77,78,79]).

### 2.2. Why Was Prolonged Activity Neglected?

The puzzled reader might now be asking why prolonged activity received so little attention in stress science, while it seems such a common-sense ingredient in stress-health link. Indeed, to our knowledge, since Selye emphasized the temporal aspect of the stress response [40], before the 1990ies only one stress researcher, Ursin [42,80] had explicitly adopted prolonged activation as a major element in his theories. Not until the late 1990ies were perspicuous attempts undertaken to put stress recovery back on the research agenda [43,44,45,81,82]. A likely cause for this neglect may be that a large part of stress science originated in experimental animal research (e.g., [41,83,84,85,86]). The use of stressors in this research was reasonable since in general, animals’ responses are temporally closely matched to the actual stressful events that caused them and not on thoughts of stressors in the past or future. Understandably, animal-based stress research did not inspire any contemplation about the typical and crucial human contribution to the prolonged stress responses: the ability to represent past and future stressful events, that is, perseverative cognition. Subsequently, this focus on stressors appears to have been further instantiated in the influential stress theories of psychiatrists Holmes and Rahe, and of Lazarus and Folkman and several other psychologists [87,88,89,90,91,92,93,94]. Even though they later emphasized subjective evaluation, the latter still pertained to stressors. Our own perseverative cognition hypothesis (see above; [48,49,50,66]) is in fact also based on stressors albeit their cognitive representation. Even recent biologically oriented theoretical models linking stress and health still start with environmental demands (see e.g., [95]), including systems-based theories such as the allostatic load model [82] and the cognitive activation theory of stress (CATS; [46]), in which stressors are conceptualized as deviations from a (changing) set point and in terms of theprobability of aversive stimuli.

### 2.3. Reconstructing Stress Theory

Thus, the centrality of stressful events in conventional stress theory appears to be largely due to influential stress theories of the previous century that use (stimulus or) stressor or stressor perception definitions of stress, or threats to resources.

#### 2.3.1. Stressor Theories

Selye’s stressors for example are “circumstances that place physical or psychological demands on an individual” [40,41]. Human stressor-science started with the recognition that life events or life changes and daily hassles could affect health [87,91]. According to Lazarus and colleagues stress is created by “environmental or internal demands that tax or exceed the adaptive resources of an individual” [88]. The “internal demands” in his definition has later been ignored by most researchers who have continued to view stressors as external forces or encounters that impinge on the body. Although Lazarus and colleagues [88,89,90] later proposed that stress is a transactional process, involving also appraisal by the person, demanding circumstances were always considered a requirement. Now, these arguments may sound convincing, but the reality is that organisms do no wait until something taxes or exceeds their resources to release a stress response. Animals (and by extension humans) do not wait for certainty about a threat in an uncertain or new situation: they immediately show a stress response, prepared to err on the side of caution. As discussed in more detail below, in many situations chronic stress responses occur without stressors or threats at all, neither present nor expected. To lift a tip of the veil, for example lonely animals and people show a chronic stress response without necessarily facing actual threats, and the same is true for people with a low social economic status. Even just having a less fit body (and thus less “safe body”) may disinhibit the default stress response.

It is very important to emphasize this again: there are many situations with prolonged activity in which *nothing actually seems to tax or exceed* resources, that is, in which no stressors are present. Through the theoretical lenses of conventional theories such as Lazarus’, these situations remain invisible as sources of stress. Even for well-known chronic stressors, such as work stress, the aspect that is most probably responsible for their prolonged stress responses, is prevented from being recognized as such: the responses are continued when workers are not actually dealing with their specific stressors or even thinking (worrying) about them. Appraisal-based theories about work stress, such as the demand-control model [94] and effort-reward model [96] involve stressful appraisal (beliefs, interpretations) but these also pertain to stressors, and do not sufficiently explain this prolonged activation associated with work stress.

#### 2.3.2. Resource Theories

There are several stress theories that focus on resources rather than stressors, for example resources such as social support [97] or person characteristics such as self-efficacy or optimism [98,99]. However, these resources are always discussed in terms of their buffer or modulator value when one is faced with stressors, such as work stressors [100], and not as stress sources on their own, for example when these resources are in short supply or absent. An exception is Hobfoll’s [101] conservation of resources (COR) theory that hypothesizes that the (threat of) loss of resources is sufficient for stress response to occur. However, when Hobfoll speaks of the environmental causes of these threats and losses, we are essentially back to *stressors* as a source of stress responses ([101], p. 516; [102]). Still, the COR theory’s acknowledgment of the non-essentiality of specific and immediate threats for the stress response, is compatible with GUTS’ central notion of a default stress response when no information of safety is present. However, there are several differences. According to GUTS, the critical resources all concern safety, directly or indirectly, and they do not need to be threatened to cause stress responses: it is enough for them to be continuously low or lost or simply not perceived due to lacking or distorted information, due to the default nature of the stress response that does not need to be “triggered”.

Taken together, to explain prolonged physiological stress responses, stress theory needs to be reconstructed to account for the default nature of the stress response. Conventional constructs are perhaps still of some use for understanding short-term responses to stressors or threats to resources but not for understanding prolonged activity: they explain not more than a fraction of it. Instead, generalized unsafety (GU) determines prolonged activity, and only perceived safety can terminate it.

How about coping strategies, that other hallmark of conventional stress theory? With the “incredible shrinking” of the role of stressors, the conventional coping constructs also becomes far less relevant. Moreover, coping strategies are typically considered *conscious* cognitive or behavioral strategies (e.g., problem solving, palliative responses, seeking social support), while the default stress response and GU are largely unconscious phenomena. We will discuss this important facet of GUTS in more detail in the following section.

### 2.4. Why the Default Stress Response Doesn’t Need Conscious Awareness

As mentioned above, some years ago we have already hypothesized that a large part of stress is unconscious ([67,68], see also [103]), and GUTS now provides several additional arguments. Although we can sometimes be conscious of and even report experiences of GU, there are strong reasons to believe that in many cases GU has no access to conscious awareness. There are at least three arguments that suggest that the default stress response to generalized unsafety (GU) in humans is largely unconscious.

#### 2.4.1. Phylogenetically Old

Firstly, the default stress response is phylogenetically extremely old, developed far before conscious awareness developed in humans. As conscious awareness is not assumed in most animals, it follows that the GU basically works without awareness. In humans, GU will often seep into conscious awareness, but this is very unlikely to be a necessary condition for GU to be maintained. This might become clearer if we investigate the evolution of the stress response itself. The stress response is phylogenetically very strongly related to that other major defense system: the immune system. However, the stress response evolved much later than the immune response against infectious agents. The immune response is essentially a defense response against threats on the proximate, molecular level, and is present in even the most primitive of organisms. The stress response on the other hand evolved to detect and defend against distal stressors far before they could harm the body and evolved in later organisms—in a way as an “extension” of the immune system. According to Maier [104,105] the immune system uses physiological systems including the SAM and HPAC system to produce energy to fight infection, and the stress response later started to use these same systems to fight off, of flee from, external stressors. One could argue that the immune system also responds in a default fashion. Innate immunity can only recognize SELF, that is, the body’s own tissue, the rest is categorized as NONSELF and attacked and removed from the body. Since *self* here equals *safe*, it follows that the immune system by default responds to everything unless it is safe (self). The later evolved stress response does basically the same: when not recognized as safe by default the response is on. The stress response uses the nervous system to tell safe from unsafe, but only much later. In hominids and perhaps some other “intelligent” animals, survival must have been promoted by the ability of the stress response to become partly conscious, for example to improve coping attempts and communication of at least parts of the stress experience.

#### 2.4.2. Continuous Responses are Hard to Perceive

The second reason that the disinhibited default stress response is (largely) independent of awareness, is that it will often be *continuously* disinhibited, because many unsafe conditions (compromised body, compromised social context, see below) are chronic by nature. However, phasic changes are much more likely to be consciously detected by the brain. Thus, a continuous emotional-visceral state typically goes undetected by conscious awareness, even in humans. As a result, the default response is likely to be misinterpreted as one’s “normal” state. It seems bitterly ironic that precisely because of the feature that makes it most harmful to health, namely its chronicity, the default stress response remains under the radar of conscious awareness, and thus undetected and sub-optimally coped with. Thus, the default response, and GU as its cause, can often not, and therefore will not be measured by self-report.

Interestingly, it is often when such a state of default stress response is interrupted that we realize its existence. For example, it is often only through the relief we feel after receiving a positive examination outcome or a benign medical diagnosis that we realize how “stressed” we actually were (On the other hand strong positive events often cause brief arousal, i.e., disinhibition of the default stress response, caused by the combination of important biological goals at stake, a possible strong action preparation (“approach”) and not unlikely a temporary uncertainty (or unsafety) with respect to these goals, in GUTS’ terminology.). An even subtler example of this is given by several studies showing that subliminal presentation with the word “relax” lowers participants’ blood pressure [74,106,107]. Were the participants stressed to start with? This finding may not be restricted to the laboratory. We truly feel less stressed after a relaxation exercise even if we “were not stressed” in the first place, let alone physiologically. This might seem a common, even trivial phenomenon. On the same account it suggests something shocking. Apparently, most of us are constantly somewhat stressed—without stressor and without being aware of it: is this the *elephant in the living room* for stress science?

#### 2.4.3. Determined in Early Life Stages with no Conscious Access

Thirdly, our default stress response is not only determined by events during our current life of which we may or may not be conscious, it is also strongly influenced by early stages in life of which we have little or no conscious awareness at all. As argued above, from birth on we slowly learn the contingencies of safety of our world. It is generally assumed that the earlier the learning experiences the stronger neuronally ingrained they are and the less accessible they are for later conscious awareness. The strongest or at least most irreversible determinants are even generated the phase before birth: our stress responses are *programmed* prenatally, genetically or in uteri, meaning that many children are born with particularly strong and persistent expectations of an unsafe world. As we will discuss in more detail below (see Compromised early safety learning and “prenatal programming”) maternal stress during pregnancy leads to more chronic stress responses in adult offspring of both animals and humans, independent of stressors (but also stronger responses to stressors), and the same is true for several personality dispositions such as neuroticism and trait anxiety that are believed to be at least partly genetically determined and thus even earlier in the process. Clearly, the contribution of these prenatal influences ensures that a significant part of our default stress response is completely unconscious and will probably remain so throughout life. An important consequence is that conscious cognitive and behavioral coping strategies that have been so central in stress theories are far less important than expected.

### 2.5. Summarizing the GUTS Principles

Before discussing in detail the compromised domains, it is important to state the formal principles of GUTS [1,2,3] here.

- **The stress response is a default response.**
  The stress response is a default response that is active when no information about safety, or no safety is perceived. When safety is perceived, the stress response is inhibited through prefrontal-subcortical inhibition. Because the stress response is “on” (activated) by default, it is already activated when there is simply no information, because by default it assumes that everything is unsafe, unless informed otherwise. Hence the name of the theory: the generalized unsafety theory of stress.
- **The basic physiological state is co-determined by perception of safety.**
  The stress response is the primary determinant of the state of the autonomic nervous system and therewith all other subsystems and organs in the body. It generally activates some (e.g., cardiovascular and some muscular systems) and inhibits others (e.g., metabolism, growth etc.), ultimately in service of fighting off threat of fleeing from it [108]. Given the stress response’s pervasiveness at all bodily levels, it follows that the basic state of any organism, of all its biological (sub)systems, at any given moment in time, is co-determined by the perception of safety. Thus, the actual physiological level fluctuates with the level of perceived safety and hence the level of disinhibition of the stress response.
- **Perceived safety is the outcome of the brain’s prediction of survival chances.**
  Perceived safety is the continuously changing outcome of a process of neurovisceral integration [109] of information from the body’s state and the environment, predicting the survival probability of the individual organism and its offspring (i.e., the passing of its genes to the next generation).
- **The stress response is largely unconscious.**
  This safety perception is nearly 100% unconscious, being a phylogenetically ancient and primary adaptive system in all animals, with only some animals, at least homo sapiens having added only a “thin layer” of conscious awareness.
- **No stressors are needed for prolonged stress responses**.
  Although the organism’s default response can also be disinhibited by “specific unsafeties”, that is, stressors (threats) or thoughts thereof (i.e., perseverative cognition [48,50,66]; see also below), in many people with prolonged default stress responses, it is likely to be far more often disinhibited *without actual stressors or thoughts thereof* In fact, only relatively few episodes of the total duration of their default stress response will be caused by specific unsafeties or thoughts thereof, while the rest of the duration of this response is determined by the unconscious perception of unsafety. Therefore, GU is a far more important cause of prolonged activity than the stress responses to specific unsafeties, that is, stressors or threats to resources, as they have been called in conventional stress theory.
- **Perseverative cognition is part of GU.**
  GU is also far more important than perseverative cognition in explaining prolonged activity. Perseverative cognition, such as worrying and ruminating [48,50], and potentially unconscious cognitive representations of threats [67,68,103], may be both *the result* of the default stress response and the cause of its maintenance. This is why even for non-pathological worriers it is so difficult to stop worrisome thinking, and why pathological as well as non-pathological worriers often do not understand what triggers worrying when the threats it pertains to are so often evidently unrealistic.
- **Safety signals for humans are primarily social and learned.**
  The safety signals that will lead to the inhibition of the default stress response are often very specific and are manifold. For social animals such as humans, cattle and many bird species, primary safety signals relate to social sources (e.g., a group one belongs to), but other safety sources may be shelter, hiding places and a surveyable environment, and there are many species-specific safety sources (see below). Many safety signals are learned, and they can become generalized, which in humans is the foundation for well-adapted individual.

## 3. Compromised Domains: Prolonged Stress Responses without Stressors

There are several very common situations in which the default stress response may be chronically disinhibited, because they share the following characteristics:

- They are associated with chronic physiological activity that is similar to a stress response;
- Their associated chronic physiological activity is not caused, or unlikely to be caused by stressors;
- They are characterized by a reduced availability of perceived safety.

We have called these situations with chronic physiological activity without stressors *compromised domains* [1,2,3].

### 3.1. Compromised Social Context

For social animals, being part of a group is a crucial important source of safety. The group provides protection for example via defensive attacks of predators or hostile conspecifics, or via “safety in numbers”, and individual animals can for example outsource the scanning for safety. Indeed, the larger the group size the lower the levels of vigilance needs to be, as evidenced in a variety of mammals such as elks, impala’s and boar, and many birds (see [110] for a brief listing). Moreover, for many species the group is not only safe but also facilitates psychobiological goals including reproduction and food. For example, less socially integrated killer whales die earlier, presumably because of less social information about the location of food (e.g., salmon) and less food-sharing opportunities in times of scarcity [111].

#### 3.1.1. Isolated Social Animals

Since the group is so crucial to safety and survival it is no surprise that social animals show chronic stress responses when isolated. Thus, a compromised social context is also an important instance of a stress response that is independent of the presence of stressors. For example, isolated rodents show behavioral stress responses as well as increased basal corticosterone secretion and increased heart activity [112]. Increased basal corticosterone levels has also been found in many other social animals when (relatively) isolated, including baboons, prairie voles, cows, and sheep, and marmosets, but less clear yet for other physiological parameters [20]. Importantly, these effects depend on several species-specific social properties (e.g., monogamous- or polygamous) and sex differences, and for example on domestication (e.g., in the case of dogs the human owner often replaces the need for connection with other dogs) [20]. Social isolation also influences disease risk and mortality in many different species [20,113], including a decreased lifespan in the fruit fly [114].

#### 3.1.2. Loneliness in Humans

Humans are also social animals. Our brain is even specialized for group living [115]. For us too, during evolution being isolated meant in most cases a rather certain and early death. Thus in humans too loneliness often goes hand in hand with chronic physiological responses that are hard to distinguish from stress responses. It is linked to chronically decreased heart rate variability (HRV) [116] and increased cortisol levels and dysregulated immunity (see [20]), with greater total peripheral resistance [TPR] and lower cardiac output (CO), in the lab (see [117]) and during daily life [22], but not with higher blood pressure [22,117]. The latter profile mentioned here, a high TPR and low CO (not necessarily accompanied by higher blood pressure), is increasingly being identified as a physiological response profile that is specific for vigilance for threat and perseverative cognition [118,119,120]. Finally, loneliness also carries increased chance of illness or early death [20,21,22,23,24,116]. Related findings across several physiological systems have been reported for low social connectedness (see review [24]). A recent study even found a dose response relationship between lower social integration and physiological dysregulation [24]. However, what causes the stress response in loneliness? Loneliness in itself not a threat. Instead, lonely people are lacking in something: a warm, supportive, safe social network, or simply perceiving sufficient friendly people around—something which is so important for social animals such as humans; call it love for a better word. Thus, lonely people show signs of a stress response, even a chronic stress response, in the absence of love, even if there is no sign whatsoever of a stressor. Again, according to GUTS this is explained by the lack of the primary source of safety for social animals, and therefore the chronic disinhibition of the default stress response.

#### 3.1.3. Low Social Status and Discriminated Minorities

Theoretically, there are other types of compromised social context, for example low social economic status (SES) and membership in a discriminated minority. Low SES, typically measured by a combination of income, education, occupation, and housing conditions, is one of the strongest predictors of morbidity and premature mortality worldwide [121,122,123,124,125,126,127], with a ten-fold or higher risk for the lowest ranking SES compared to the highest, independent of any differences in material deprivation [126]. Low-SES people show a chronic low HRV [109,128,129,130,131], and increases in other major peripheral biological parameters, including blood pressure, sympathetic activity, and disruptions in HPA axis regulation (see for an overview [132]). Only roughly one-third of the health and mortality risks are explained by life style factors. Although low SES people experience many stressors, the strongest explaining factors are low self-esteem (internalized inferiority; [126]), lack of control, subjectively low SES (the feeling where one stands relative to the others in the hierarchy; e.g., [126,133]) and lack of social capital the lack of important social relations at the level of the community [127]: all factors that are not discrete stressors, but seem to point at reduced (social) resources. Thus, being relatively lower on the SES ladder seems to be a compromised situation and associated with generalized unsafety. Several studies support this idea, for example showing that adolescents from low-SES families interpret ambiguous situations as more threatening, a tendency that persists in adulthood [134].

A well-known example of membership in a discriminated minority with serious health consequences are African Americans (AAs) in the US. AAs have lower life expectancy than their compatriots [135,136], and it is believed that discrimination significantly contributes to this difference [137]. AAs lifetime burden of perceived discrimination and discriminatory harassment and/or assault is associated with higher blood pressure, especially ambulatory and nighttime diastolic blood pressure [138] and higher levels of inflammatory markers (see [139]). Again, since it is unlikely that these chronically enhanced physiological effects are due to actual discrimination-related stressors alone, or to continuous conscious worrying about it, it seems more likely that for many AA’s daily life is unconsciously perceived as less safe than for others.

#### 3.1.4. Other Compromised Social Contexts

Furthermore, many aspects potentially make modern society a socially unpredictable and therefore less safe place, such as the increase in ambiguity of norms, the rapidly changing rules and loss of (shared) rituals, that will not be discussed here because of space limits. Also, there are elements of social compromises in other chronic stress situations, such as work stress, financial debts, and the non-natural urbanized environment, which we will discuss below.

Finally, it should be noted, that the effects of loss of social resources can occur already at low levels of deprivation. According to Coan and colleagues [140], at baseline before an experiment, the brain looks more “at rest” when other people are present than when alone. Thus, being alone may be an erroneous baseline for stress experiments, ignoring the social nature of human beings, yet it is used by far in the majority of experiments (cf. Coan’s Social Baseline Theory; [140]). In a particularly intriguing study by Eisenberger and colleagues [141] participants who were “excluded” in a rather silly computerized ball throwing game showed neural activation localized in a dorsal portion of the anterior cingulate cortex (dACC) that is implicated in the affective component of the physical pain response. According to the authors the survival value of social bonding is so important that is uses the brain’s pain system to stimulate us to seek social inclusion.

### 3.2. Compromised Early Safety Learning and “Prenatal Programming”

As mentioned in the beginning, from birth on animals and humans gradually learn to recognize safety, especially in the early phase of life. After leaving the safe uterine world, babies show a disinhibited default stress response: initially the world appears completely unsafe for them. Interestingly, newborns do not yet show the cardiac deceleration that accompanies the orienting response toward new stimuli seen in adults: they initially keep on responding with heart acceleration (i.e., a defense response) to new information right into their 6th month [142]. Slowly the newborns learn to predict the safety signals around them: the conditions under which hunger and thirst are satisfied, when affiliation needs are being met, reflected in an increasing HRV [116]. In the course of life their brains learn the increasing complexity of the contingencies of safety and their generalizability toward new people, new situations and environments, and thus when and where to inhibit the default stress response (see our discussion elsewhere: [1,2]). Unfortunately, much can go wrong during this process, often as the result of early life stress or emotional neglect. Early adversities, especially physical or sexual abuse, emotional neglect, and household dysfunction may disrupt the young child’s safety learning, including the generalization of a sense of safety: “the world is a safe place”. Early life stress is associated with chronic lower HRV [26,27] that continues into adulthood [28,143] and with increased risk for organic disease [144,145,146], even in a dose-response fashion [30]. Again, since these chronic levels cannot be due to actual stressors, according to GUTS they reflect a default stress response that is inefficiently inhibited due to a generalized perception of unsafety: “the world is an *unsafe* place”. Indeed, maltreated children seem to perceive more threat in ambiguous situations [147]. Emotional neglect or maltreatment is also believed to lead to stable psychological characteristics such as low levels of emotional awareness [103] and alexithymia, which are also predictors of ill health [103,148].

No less grim is the situation in the case of even earlier, *prenatal* determinants of the stress response. The fetus’ brain appears to make fixed predictions of safety based on the mother’s experience: it learns already in utero that the world is an unsafe place. Thus, maternal stress during pregnancy leads to more behavioral and physiological chronic and acute stress responses in adult offspring of both animals and humans (e.g., [31]), at least for the endocrine and immune responses ([32,33]; and even distortion of intestinal microbiota [34] but not for cardiovascular responses [149,150,151]). Taken together, children of mothers who have been stressed during pregnancy tend to show chronically enhanced stress levels through adult life. Note that the born baby is not programmed for certain stressors, but for a lower safety expectancy!

Even less reversible, if at all, are the expectations concerning unsafety that have already been printed in the *genetic* code: we differ in our hereditary dispositions with respect to the default stress response. These prenatal influences are partly reflected in personality characteristics such as neuroticism, trait anxiety, trait anger and hostility, and repressive coping style that at their core carry a strong expectation bias of unsafety, similar to anxiety disorders (see above). All of them are associated with prolonged stress responses, even in the absence of stressors [47], and they are predictors of somatic disease [35,36,44,148,152,153,154,155]. We speculate here that the high prolonged physiological activity found in emotionally unaware people (e.g., in alexithymia, the repressive coping style and low emotional awareness) is not recognized due to its constancy (see above: “Continuous responses are hard to perceive”).

### 3.3. Compromised Physical Environment

There are many examples of animals that seem “stressed” without actual stressors present. All that is often needed is the presence of another strange animal or human, an owner’s unusual behavior, a clean litter box or simply nowhere to hide, for stress levels to increase [156,157,158,159,160,161]. For example, if a cat a place to hide in a new environment, its “behavioral stress levels” recover faster than those without a hiding place (e.g., a cardboard box; [162]), but not when housed in pairs or groups rather than alone [163]. Other than humans and other highly social animals, most members of the genus *Felis* are solitary. Their perceived safety relies relatively strongly on properties of their core territory, including its familiarity and the presence of hiding places (e.g., a hole, a tree). Also, cats perceive a new environment as more potentially threatening than many other animals do [164]. It has been known for a long time that novelty evokes physiological stress responses (e.g., [85,165,166]). Stress responses in novel situations and/or a lack of hiding places are also examples of physical contexts with stress responses but without stressors, and thus merely unsafe contexts. We call them compromised physical contexts. In fact, novelty, as a situation without familiar information, is a prototypical situation for the default stress response. It is likely that novel environments are even more stressful for territorial animals such as cats [164] than for social animals.

#### 3.3.1. Distorted Information

Further, there are many ways in which physical environments can obscure information needed for safety, including distortion of information, for example by darkness, dense fog, or loud noise. Continuous noise such as road traffic noise leads to chronic physiological stress activity [167,168,169], also during sleep [167], and increased risks for cardiovascular disease [170,171]. Noise has often been used in stress experiments, and we take it for granted that loud, sustained noise can be used as a stressor. However, following GUTS we argue that at least part of the stress-evoking effect of continuous noise originates from the fact that it blurs information about possible impending dangers, including direct sounds of for example a predator. In nature, rain or hard blowing winds may have these effects. Thus, with noise a physical environment that on itself is safe may become less so. Conversely, too little sound may sometimes also mean unsafety such as the warning signal of birds silencing because of a looming predator (e.g., [172]). It was also found that human babies are not only stressed by too much noise but also by too little sound [173]. Purportedly, for babies on-going human sound confirms the closeness of protective others, the safe group, and so this links in with compromised social context as discussed above.

#### 3.3.2. Nature Versus Urban

An intriguing, and more general example of a potentially compromised environment is our own human urban or otherwise anthropogenic environment. There is a growing literature on the direct health promoting effects of nature—that is, being in nature, or experiencing nature, as opposed to using it for exercise or for its direct effects such as fresh air, “natural” foods, etc. (see e.g., [174,175]). This literature strongly suggests that exposure to natural, be it green or blue (i.e., water; [176]), as opposed to urban environments reduces stress (anxiety, depressive feelings; [177]) and increases HRV and lowers levels of heart rate, cortisol, and blood pressure [174,178]. In general, the preference for and the health promoting effects of natural environments as opposed to urban ones is upheld when controlling for a host of possible confounding factors such as socio-economic status and levels of exercise, or other adverse or potentially noxious aspects of urban environments, such as noise, smells and crowdedness (see [174,175]). Importantly, although the majority of studies focused on stress recovery, many studies have established that simply being in nature, or proximity to nature, or being exposed to natural stimuli is sufficient for the health promoting effects [176,177,178,179,180,181,182,183]. For example, simply viewing natural pictures versus urban already increased HRV [179]. Moreover, positive effects of being in nature are not necessarily mediated by awareness [184].

Of the many theoretical explanations that have been provided—and criticized—two types of explanations stand out in the context of this paper. Firstly, natural stimuli or perhaps even specific landscapes may evolutionarily have provided signals of safety (hiding places, lookouts, information about escape routes etc.) and resources such as food and water. While the idea of innate preference for specific landscapes may be attractive, it is at odds with the wide variety of landscapes in which our successive ancestors have lived in [185]: savannah, jungle, deserts, mountains, snow- and ice sheets, coastal and river areas. Yet, while no specific landscape might be “wired in”, more abstract environmental aspects that facilitate safety perception, food gathering etc. might have been so. Secondly, natural environmental information would fulfill innate desires to understand and explore, and we are evolutionarily adapted for processing natural stimuli [174,186]. Indeed, there is abundant evidence showing that we are better at navigating, orienting, and locating in natural surroundings [186]. To give some examples out of many, we have better memory and an attentional priority for natural information [187,188,189,190,191], and we are far more accurate at noticing changes in nonhuman animals than for vehicles, despite the far greater harmfulness of the latter in modern life [192].

Together, these explanations are just one step away from the GUTS position: urban environments do not provide sufficient information about safety, and additionally, our perceptual system is shaped by ancestral selection pressures to be specialized in natural information. Put in another way, in a natural context much more information is code-able on a dimension safe-unsafe, while in an artificial, urban context much more will be coded as ambiguous. Thus, because of a lack of genetically preferred information urban environments do not provide enough information and thus the default stress response remains disinhibited in them.

#### 3.3.3. Urban Environment as Territory of Unknown Others

There is an intriguing alternative possibility, which ties in with the notion of compromised social context. The built environment, especially urban, tends to be largely the property of unknown others (strangers, companies, impersonal public authority). Although our species is not as xenophobic as most other primates (see [193]), meeting strangers or being in the territory of others is likely to disinhibit the default stress response at least partially. On the other hand, cooperation with—and thus trust in—strangers is one of cornerstones of the evolutionary success of our species. Yet, in the absence of previous experience with a stranger, humans rely on signals of positivity in establishing trust (see [193]), and thus perceive safety. These signals are partially innate and partially learned. Importantly though, when an environment offers limited or absent communication with strangers, as is the case in many urban areas, this trust or safety cannot be fully established, which might result in a maintained default stress response to some extent. In fact, in large cities, by far the greatest part of the perceived environment is owned by unknown others. In other words, many of us live to some degree in an alienated world with little means to perceive the safety granted by interhuman trust. Conversely, one’s own little street or village—however anthropogenic it is—might convey a feeling of safety and help keep the default stress response inhibited. Similarly, natural environments provide a place that is either *not* obviously owned by unknown others, or “owned by all” or by “our group”.

Therefore, is just being in an urban environment enough to—at least slightly—release the default stress response? To our knowledge no studies have yet provided direct comparisons of chronic physiological activity levels between urban areas “owned by others” and areas “owned by known and trusted others” or “by no one”. Until this is tested, we hypothesize that, just as being alone, being in an urban environment is an erroneous baseline condition (see above: social baseline theory [140]).

### 3.4. Compromised Bodies

#### 3.4.1. Compromised Animal Bodies

A capable, clean, and healthy body is a primary asset to preserve safety. For example, birds appear stressed if they cannot wash their wings. When a starling does not have access to water to clean its wings, it becomes more vigilant and more aware of danger. The starling who cannot wash its wings, even when they are not necessarily dirty, seems therefore to show a stronger stress response, while there is no sign of an actual stressor [194]. This is most certainly not an isolated case, but likely to be the same for many other birds. Other birds, such as zebra finches show a higher level of the stress hormone corticosterone if they have not been able to bathe for a while [195]. This makes sense, for without water to wash one’s wings, fleeing when danger approaches may become far less effective. Being more alert when there are no washing opportunities is therefore highly beneficial for survival. The more vigilant birds were the ones who could pass on their genes.

For the same reason, many animals use dust baths instead of water baths to maintain healthy feathers, skin, or fur, and for example chickens when deprived of dust bath opportunities (e.g., sand) show behavioral and physiological stress (i.e., increased corticosterone) responses (e.g., [196]). Animals that became more vigilant in these compromised environments were the ones who must have passed on their genes.

Independent of environmental washing opportunities, a dirty plumage or skin exposes the animal for a more direct lack of safety: a compromised ability to fight or flee. This is likely to be the reason dirty and badly cared for animals look more stressed, as farmers can tell. However, there are many more ways that a body can become compromised.

For example, a squid that has lost part of one of its eight legs reacts to a small neutral object (e.g., a wire) with a stress response. When presented with such an object it retreats quicker, hides, flees quicker or squirts a cloud of ink quicker than normal [197]. So even being mildly handicapped makes a squid much more alert even when there is no immediate stressor.

Another, quite different example. Rats that were experimentally made obese showed more stressed behavior (anxiety and depression-like behavior) and higher corticosterone, independent of a stressor. In fact, their stress levels were comparable with non-obese rats during a chronic stressor, and when stressed themselves their levels did not even go up, suggesting that their stress levels had already reached a ceiling by being obese alone [198].

#### 3.4.2. Obesity, Low Aerobic Fitness and Old Age as Compromised States

To our knowledge, in humans, missing bathing opportunities, dirty skin and missing body parts have not yet been linked to chronic stress responses. However, obesity in humans has been associated with chronically enhanced physiological levels such as increased heart activity, high blood pressure and endocrine increases that are similar if not equal to a chronic stress response. This is also found in other “compromised” bodily states, such as low aerobic fitness and old age [199,200,201]. All these conditions carry significant health risks. Still they are seldom considered to be direct causes of stress responses. According to GUTS, animals and humans in these conditions are stressed. Thus, irrespective of other biological mechanisms that cause increases in physiological activity, the default stress response in these physical conditions is not being fully inhibited because, through millions of years of evolution, they carry with them a less adequate fight or flight response. In other words, they are “not optimally resilient” bodily conditions. An older or less fit body reacts slower to its potentially dangerous surroundings, just like the dirty birds or the handicapped squid, which means their world is less safe. With every extra pound or drop in fitness, the less safe your world is. High alertness was therefore key for survival. For millions of years, our world was a predatory and dangerous place to live in, each day could be your last. Therefore, our compromised body maintains the default response, at least to some extent, because safety cannot be fully perceived, or could not be fully perceived by our ancestors. Those who kept their stress response disinhibited when their bodies were—in whatever way—compromised were the ones who must have passed on their genes.

#### 3.4.3. Other Compromised Body Examples

The examples above were chosen because these conditions do not involve intrinsic distress, or in other words, they do not act as a threat themselves. This is the case in numerous other potentially long-term bodily conditions such as fatigue, pain, other medically unexplained symptoms, sleep deprivation, illness, persistent subclinical inflammation, or—more recently explored—deficient intestinal microbiota (dysbiosis [202]), heat, cold, or enhanced physiological load by substances such as alcohol, caffeine or drugs including pharmaceuticals, and in some parts of the world hunger and thirst. According to GUTS, in all these conditions the default response will be disinhibited, at least to some extent, because safety is not fully guaranteed anymore. Theoretically this response will come on top of that caused by the distress of these conditions because of the conditions themselves being perceived as threat), *and* the possible increases in partly the same physiological systems caused by the various biological processes underlying each of these conditions, the detailing of which would require much more space than allotted for this paper.

### 3.5. Compromised Context of (Presumed) Stressors

It is a truism to say that stressors are unavoidable in life, and all of us, even those with a warm and close social network, with superb safety learning in early life, and living in a safe and sound environment with lots of green and blue, must face the fact that there are always stressors on the horizon. Illness or pain will never be abolished, accidents will keep on threatening us all the time, the nasty boss is unlikely to change, just as lions will always be present in the life of antelopes. The “secret” is to keep the exposure to these stressors outside of their actual occurrence at a minimum, which includes the perseverative cognition about them. However, even then, according to the “reversed” stress theory GUTS, as discussed in previous articles [1,2,3] a considerable part of prolonged stress responses occur in the *context* (or environment) where people have previously experienced stressors, rather than during these stressors—or during the conscious perseveration about them for that matter. This is also true for contexts of *presumed* stressors, that is, unrealistic threats.

First, the default stress response can theoretically remain disinhibited in the context in which a threatening stimulus (stressor) occurred earlier or with which a stressor is associated via *context conditioning*. Earlier [2] we discussed examples of this form of “compromised context” for work stress, the stressor of financial debt, and care giving for a spouse with Alzheimer’s. Stress responses can be conditioned to the wider environment of these stressors, that is, contextually conditioned [203], including such modern environments such as email and mobile phones. Since both the stressful incidences that comprise these stressors and perseverative cognition about them may be limited in time, the considerable disease risk of these stressors [53,59,65,204] may be largely explained by default stress responses to these wider associated environments.

The situation is even worse for chronic anxiety, which also carries a high organic disease risk [35,36,37] and which is characterized by a continuous failure to perceive safety where others do so. A very common disorder such as generalized anxiety disorder (GAD), forms the most extreme example of a compromised domain of context: GAD patients worry in virtually every context although most contexts are safe, making them extremely contextually conditioned [2,203]. Social anxiety disorder (SAD) too is very common and is maintained even though by far most social interactions are non-threatening, and even objectively (“for others”) positive.

The compromised contexts mentioned in this paragraph have all been found to be characterized by chronically low HRV or increased levels in other physiological stress responses, or both, in most studies (see [2]), also in absence of the [presumed] stressors themselves. Even in chronically anxious people such as GAD and SAD patients it is unlikely that they think truly incessantly, either consciously or unconsciously about their fears, and thus neither these fears (concerning presumed stressors) nor perseverative cognition can explain their continuously increased physiological response.

An easily missed category of compromised context is that of life-threatening or serious disabling disease. For example, for cancer patients an astonishing number of contexts seem to become unsafe [205]: food information, TV and other media because of medical shows, even abstract art in which they might see uncontrollable tissue growth. Low HRV predicts mortality in cancer patients (see reviews [206,207,208]) as well as in terminally ill patients in hospice care units [209,210]. Their low HRV might not only be caused by realistic worries about the mortal threat—about which not a lot can be done—but also by contexts that they (partly unconsciously) perceive as unsafe and to which perhaps something can be done.

## 4. Conclusions

In this paper, we have argued that the default nature of the stress response, which is inhibited by perceived safety, and disinhibited when no safety is perceived, has several consequences for the science of stress and health. First, it necessitates a conceptual reconstruction of conventional stress theory, accounting for the primary role of safety rather that stressors or threats. Second, following GUTS, an astonishing array of situations and conditions (called “compromised domains”) are unveiled in which chronic stress responses occur, but with no threats or stressors—and of many of these situations and conditions significant organic disease risk have already been documented. Importantly, it should be noted that the prolonged stress responses seen in several compromised domains discussed above are *direct* effects, which is crucially different than the capacity in several of them to act as *modulators* of the effect of stressors on physiology or disease, for example (lack of) social support or (lack of) nature, (old) age and (lack of) aerobic fitness. Another important point to make here is that obviously, hardly any of the compromised domains will exist in isolation: they most likely occur together, often with more than two at a time: old age with loneliness and low fitness, the latter with obesity, fatigue in cancer, etc. Whether this co-occurrence increases the default response in an additive or interactional fashion, or not at all, is a matter of future study.

One important question has not yet been addressed, and that is how one can increase safety perception, and therewith help to tonically inhibit the default stress response. Although it is beyond the scope of this paper to address all potential ways to do this, we will mention some suggestions. A logical way would be to change compromised conditions where this is possible, such as increase social integration or social competence, or both, promote exposure to nature, reduce obesity and improve physical fitness.

From a clinical stress-management point of view, the GUTS principles suggest that when treating patients suffering from stress-related disorders (anxiety, mood disorder, burnout), therapists should focus on factors that promote the (unconscious) perception of safety rather than on stressors. Clinical stress management tends to use stressor-based or “reactive” theories: patients need to learn new cognitive or emotional coping skills to deal with upcoming stressors that are believed to trigger their stress responses. From this standpoint the new skills can only be practiced and learned during or around stressful encounters. On the contrary, according to GUTS the default response is (nearly) always disinhibited in these patients, and actual stressors or thoughts thereof account only for a small portion of it. The focus should not be on stressors but on a much broader window of opportunities for intervention, and therapists can find in GUTS a theoretical rationale for motivating their patients to engage in activities that generally promote feelings of safety. In fact, every moment in daily life can be used to (re)learn safety and expand the patients time window of prefrontal inhibition of the stress response, by (a) improving; (b) finding or (c) creating sources of perceived safety. Improving safety sources (a) can be obtained by enhancing existing safety sources such as the social network, the body’s fitness, or environmental resources such as nature.

Furthermore, new sources of safety can be found (b), but not by using self-report since perceived safety is thought to be largely unconscious. Instead, for example real life biofeedback could be used to identify such sources (notably HRV as an index of prefrontal-subcortical inhibition). A promising new ambulatory physiological technique, called “additional HRV” [79,211] consists of identifying—in daily life—HRV changes that are corrected for metabolic demands from physical activity, and thus “psychogenic”. By using this technique, it is possible to detect episodes of “psychogenic” HRV increase that are likely to reflect unconsciously perceived safety, and actively expand these periods. Such episodes can also be artificially created (c) and expanded, by using “on the spot” (ecological momentary) meditation or relaxation procedures [212], or a novel technique, transcutaneous vagal nerve stimulation (tVNS [213,214,215]) which is a new non-invasive and safe method to stimulate the vagus nerve via the concha part of the ear that is innervated for 100% by the auricular branch of the vagus. Several small trials suggested that t-VNS has positive effects on self-reported mood [214] and increases HRV [215]. Crucially, these meditation, relaxation exercises or VNS-stimulation can be administered *in stressor-related contexts*. The rationale behind such a treatment is that across time the stressor-related context, to which low HRV has become a contextually conditioned response ([203]; see above), will become associated instead with an increase in HRV: *the brain learns that the contexts are safe*. This “enhanced form of exposure” is expected to work based on the mechanism that underlies exposure therapy in phobic anxiety: by repeated exposure to feared situations (i.e., the context is CS+), bodily fear (stress) responses habituate and the situation becomes associated with the decreased stress response. Stress responses in stressor-free contexts can be “unlearned” in a similar way, because these responses are in fact as “unrealistic” as the fear response in phobic anxiety. All of this does not mean that learning to cope with stressors is useless. Even if coping with one stressor does not abolish all the stress response to it, the experience of having control may generalize to new situations, leading to “immunization” to subsequent stressors by “turning on” the ventromedial prefrontal cortex [5].

Overall, GUTS identifies and explains far more stress-related physiological activity that is responsible for disease and mortality than current stress theories. Furthermore, it does so in a more parsimonious fashion, by using an overarching explanatory principle—generalized unsafety and the default stress response—than the various biological pathways for all these conditions that are more commonly used (and that still may be partially accurate). As such, GUTS provides a revolutionary evolutionary perspective on contemporary health problems related to among others obesity, depression, loneliness, the aging society and the anthropogenization of the environment.
